# The paradox of enrichment in phytoplankton by induced competitive interactions

**DOI:** 10.1038/srep02835

**Published:** 2013-10-03

**Authors:** Jerrold M. Tubay, Hiromu Ito, Takashi Uehara, Satoshi Kakishima, Satoru Morita, Tatsuya Togashi, Kei-ichi Tainaka, Mohan P. Niraula, Beatriz E. Casareto, Yoshimi Suzuki, Jin Yoshimura

**Affiliations:** 1 Graduate School of Science and Technology, Shizuoka University, 3-5-1 Johoku, Naka-ku, Hamamatsu, 432-8561, Japan; 2 Mathematics Division, Institute of Mathematical Sciences and Physics, University of the Philippines Los Baños, College, Laguna 4031, Philippines; 3 Department of Mathematical and Systems Engineering, Shizuoka University, Hamamatsu, 432-8561, Japan; 4 Marine Biosystems Research Center, Chiba University, Uchiura, Kamogawa, Chiba 299-5502, Japan; 5 Graduate School of Science and Technology, Shizuoka University, 836 Ohya, Suruga-ku, Shizuoka, 422-8529, Japan; 6 Department of Environmental and Forest Biology, State University of New York College of Environmental Science and Forestry, Syracuse, NY 13210 USA

## Abstract

The biodiversity loss of phytoplankton with eutrophication has been reported in many aquatic ecosystems, e.g., water pollution and red tides. This phenomenon seems similar, but different from the paradox of enrichment via trophic interactions, e.g., predator-prey systems. We here propose the paradox of enrichment by induced competitive interactions using multiple contact process (a lattice Lotka-Volterra competition model). Simulation results demonstrate how eutrophication invokes more competitions in a competitive ecosystem resulting in the loss of phytoplankton diversity in ecological time. The paradox is enhanced under local interactions, indicating that the limited dispersal of phytoplankton reduces interspecific competition greatly. Thus, the paradox of enrichment appears when eutrophication destroys an ecosystem either by elevated interspecific competition within a trophic level and/or destabilization by trophic interactions. Unless eutrophication due to human activities is ceased, the world's aquatic ecosystems will be at risk.

Increasing the supply of energy and nutrients to environment and natural ecosystems has been apparent in recent years. This can be attributed to the increasing demand of the human population for food and other necessities for daily living. Such human practice can be catastrophic according to Rosenzweig's paradox of enrichment^1^. In his seminal paper, he showed that eutrophication destroys trophic interactions (exploitation) in various two-species ecosystems, e.g., predator-prey. He showed that increasing supply of limited nutrients or energy destabilizes the steady states of ecosystems. Trophic cascades are recognized in aquatic ecosystems, e.g. lakes and ponds^2^,^3^. Many studies are carried on food web and trophic cascades and the main results of these trophic interactions are often the destabilization of ecosystems^1^,^4^.

Many limnological studies also reveal that the paradox of enrichment also appears in a single trophic level^5^,^6^,^7^,^8^,^9^. In freshwater ecosystems such as lakes and ponds, the paradox of enrichment can be clearly observed during eutrophication. This process of enrichment, brought by increased nitrates and phosphates, causes one algal species to bloom and destabilized the steady-state of the system resulting in a few or one phytoplankton species, e.g., Sanaru Lake at Hamamatsu city in 2008^6^. However, in natural waters with minimal nutrient flow from human activities and other sources, existence of several competing plankton species is extremely common^7^,^8^,^9^. Since many phytoplankton species thrive in freshwater lakes, one might think that competition for the same resources, such as space and nutrients, might be severe. However, in an environment with a sufficiently large but limited space, competition between different species of phytoplankton is very unlikely because of the vast space between very small algal species with low population density. As a result, competition between different species is weaker compared to intraspecific competition.

In this paper, we construct a lattice explicit model of multi-species competition (multiple contact process) with both local and global interactions. This model replicates the competition for space (sunlight) of different phytoplankton species under varying nutrient conditions. Under local interaction, an individual reproduces its offspring only in an adjacent vacant site. In contrast, under global interaction, it reproduces its offspring in any vacant site in the entire lattice space. Under local interaction, this model becomes “lattice Lotka-Volterra model (LLVM)”^10^,^11^ and under global interaction, Lotka-Volterra model, also called mean-field theory^9^. This lattice model was first studied by Tainaka^10^,^12^,^13^ using a system of three competing species who later concluded that the dynamics is highly dependent on the size of the lattice and pointed out other interesting spatial patterns at a phase transition. In 1992, Matsuda, Ogita and Sato studied LLVM for more generality compared to Tainaka's study which is limited to interspecific interaction and without vacant site^11^. Some of their results are the critical condition for population sustenance and stationary state against mutant invasion^11^.

In this study, competition under local interaction simulates the lake or pond ecosystem with no water disturbance while the global interaction replicates complete water mixing. In our model, Gause's Law of competitive exclusion of all but one species is expected at the equilibrium state of both local and global interaction because the difference in birth rates is inevitable^10^,^14^. Furthermore, we assume that the variable birth rates (cell division rates) phytoplankton species are directly proportional to the nutrient concentration of the system. We investigate the persistence of species in ecological time scale in terms of spatial competition avoidance and nutrient availability. We show that the paradox of enrichment appears in the absence of trophic interactions. We should also note that this is the second application of the LLVM with ten species to aquatic ecosystems and the first application to the paradox of enrichment, since almost all other models of LLVM deals with a few species (usually 2–3 species, much less than 10 species).

## Results

**Figure 1:**
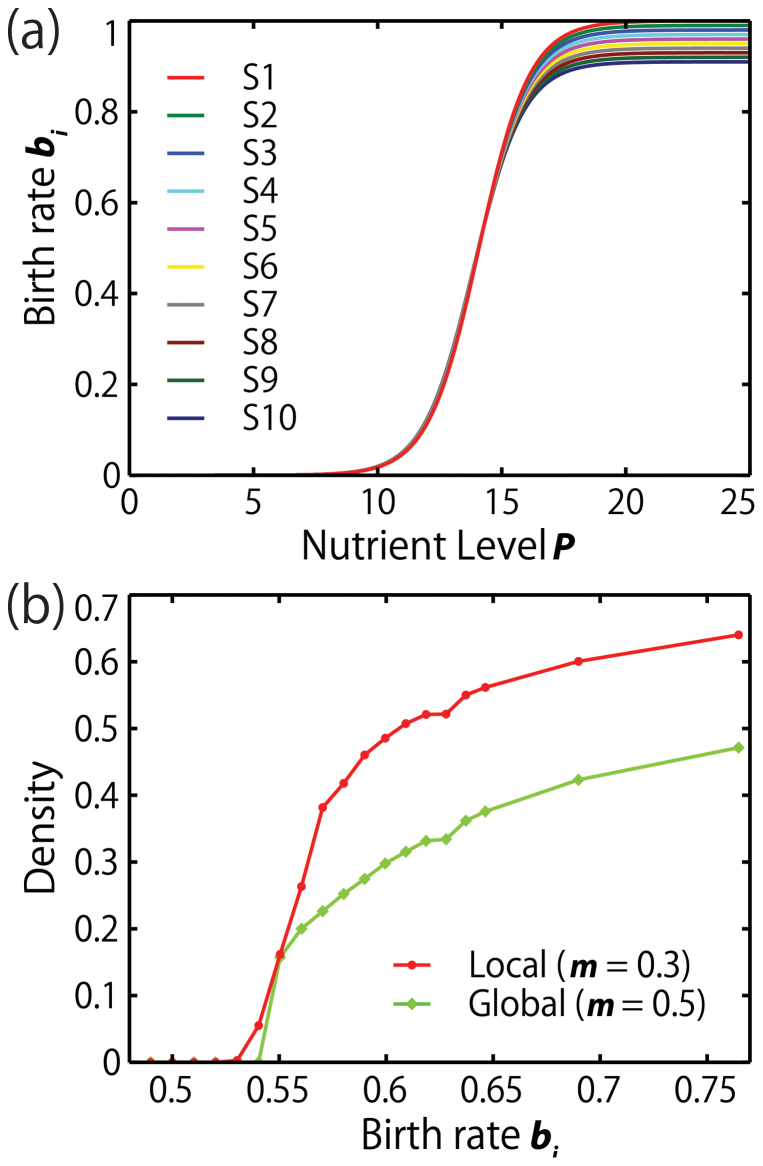
Birth rate profiles along nutrient level and density profiles against birth rates in ten phytoplankton species. (a) Birth rate of the ten phytoplankton species vs. nutrient level used in the simulations processes under local and global interactions. The study assumed that the birth rates follow a logistic function which is dependent on the nutrient availability of the aquatic ecosystem. (b) Steady-state density vs. birth rate in a single-species lattice ecosystem under local and global interactions. Different death rates are assigned to local and global interactions which are kept at *m* = 0.3 and *m* = 0.5, respectively. For both interactions, the steady-state density is estimated at around 20,000 Monte Carlo steps. The lattice size is 1000 × 1000. The threshold value for positive density for local interaction is *b_i_* ≈ 0.53 (*b_i_* ≈ 0.54 for global interaction which is slightly higher because of the higher mortality rate).

The birth rate of a species at low nutrient levels should be small with almost no variability and increase with eutrophication, since algal growth in natural oligotrophic waters is slow^15^,^16^. Since birth rates increase proportionally as nutrient level rises, they are set to be logistic functions of the nutrient level^17^ (Fig. 1a and Eq. (3)). The nutrient level *P* represents the limiting nutrients required for growth and reproduction, e.g., ammonium^18^,^19^ (Eq. 3). The mortalities of all species are kept constant at *m* = 0.3 for local and at *m* = 0.5 for global interaction, so that the overall growth rate *r* = 0 for all species below birth rate *b_i_* = 0.53 and the density profiles are comparable between local and global interactions (Fig. 1b).

**Figure 2:**
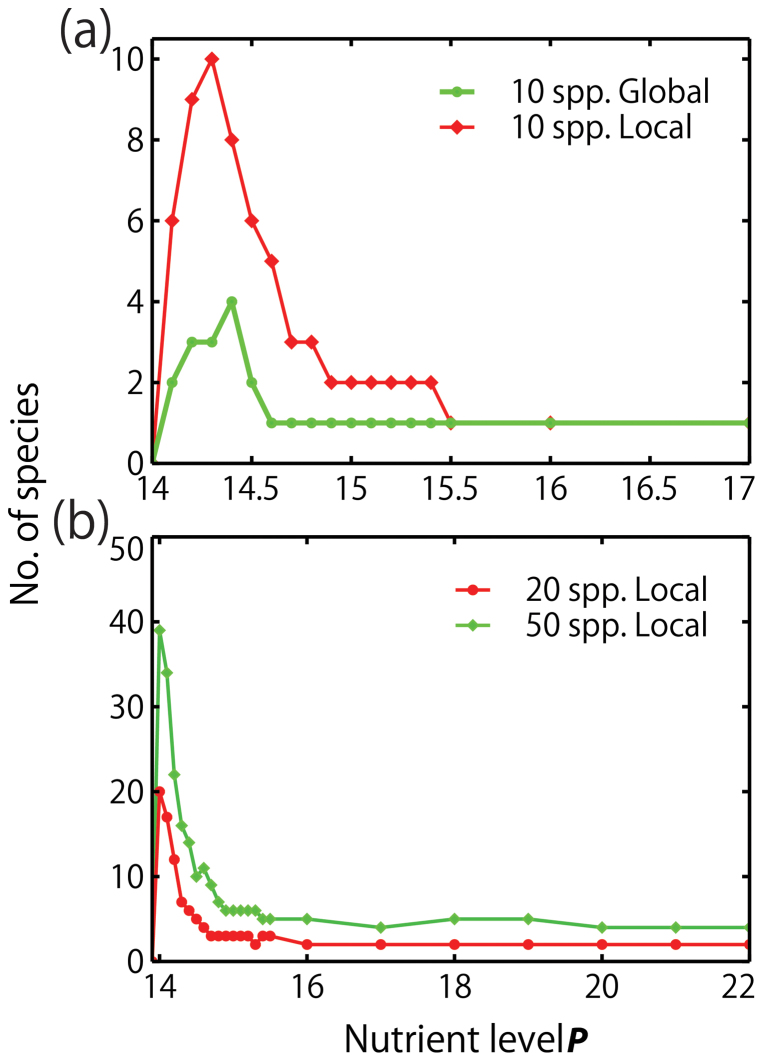
Species diversity after 20,000 time steps plotted against nutrient level *P*. (a) The number of surviving species out of ten species under local and global interactions. (b) The number of surviving species out of 20 and 50 species under local interaction.

To run the simulation quicker, we place a slight difference in birth rate among all ten species (See Eq. 3 in Methods and Fig. 2). Because high variation is expected in species-specific growth rates in eutrophicated waters, the maximum birth rates are set to be different among species (Fig. 1). We also place tradeoffs among species such that those with higher maximum birth rates at high nutrient condition have lower birth rates when nutrient is poor (close to *r* = 0), since no species are almighty in nature^15^,^16^,^18^,^19^,^20^,^21^.

We simulate all nutrient conditions from the lowest nutrient level that will allow at least one algal species to survive. We conduct simulation for both local and global interactions with an ecologically long time scale of 20,000 Monte Carlo steps. We are not interested in the equilibrium at mathematical infinity, since life on earth is finite including life in lakes and ponds. This temporal aspect of coexistence in a finite time horizon is different from the traditional studies of coexistence based on equilibrium analysis^22^.

**Figure 3:**
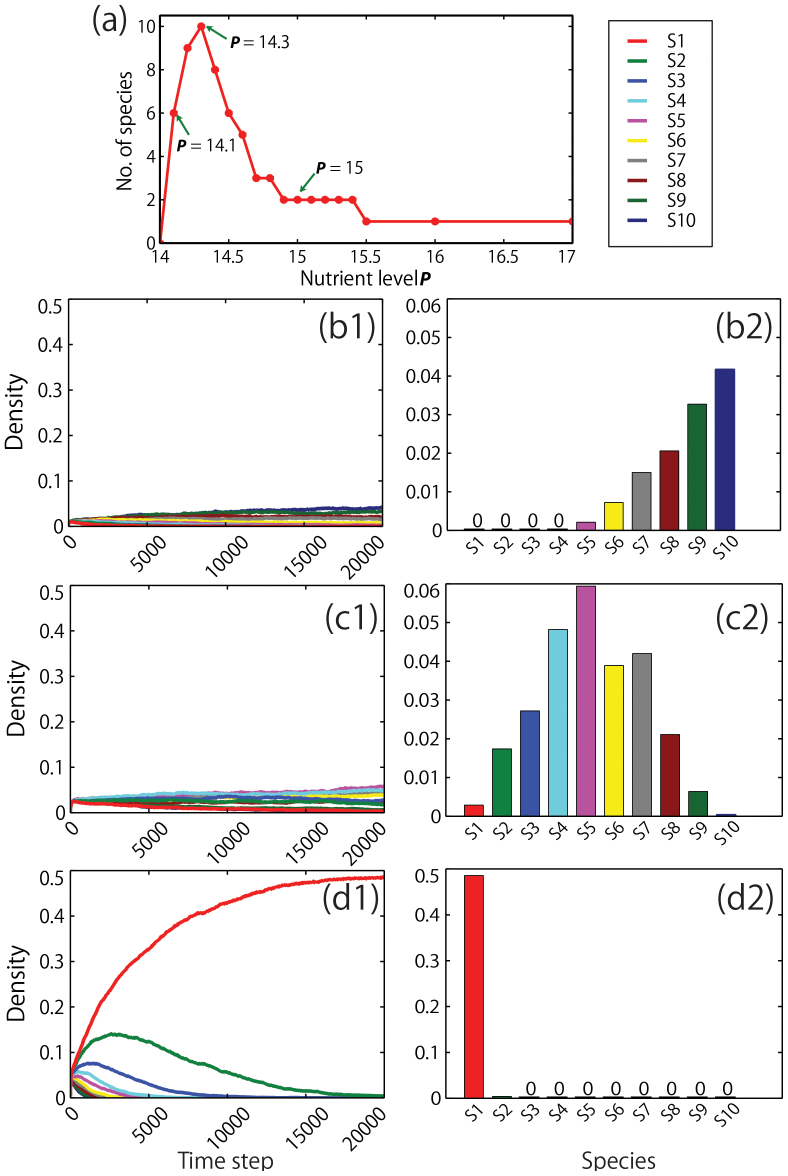
Species diversity vs. nutrient level *P* and population density of phytoplankton species vs. time with the densities under local interaction. (a) Species diversity vs. nutrient level *P* under local interaction (arrows indicate the nutrient levels shown in the following figures). (b1) Population density over time and; (b2) densities at *P* = 14.1, showing very low densities (with maximum density of about 0.04) but with high diversity of species. (c1) Population density over time and; (c2) densities at *P* = 14.3 (diversity peak), showing very low densities (with maximum density of about 0.06). (d1) Population density over time and; (d2) densities at *P* = 15, showing a very high density but with only two species surviving after 20,000 time steps.

**Figure 4:**
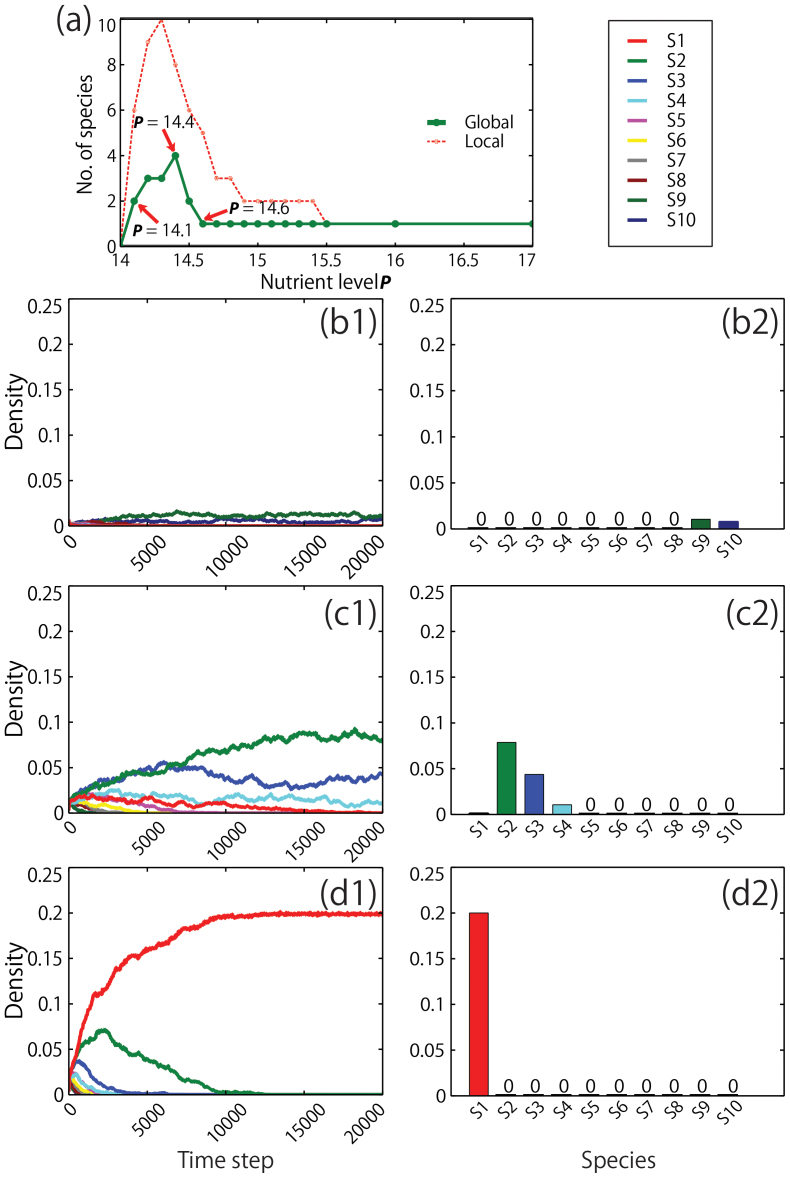
Species diversity vs. nutrient level *P* and population density of phytoplankton species vs. time with the densities under global interaction. (a) Species diversity vs. nutrient level *P* under global interaction (dashed plot is local interaction and arrows indicate the nutrient levels shown in the following figures). (b1) Population density over time and; (b2) densities at *P* = 14.1 showing very low densities (with maximum density of about 0.01). (c1) Population density over time and; (c2) densities at *P* = 14.4 (diversity peak), showing very low densities (with maximum density of about 0.07). (d1) Population density over time and; (d2) densities at *P* = 14.6, showing a very high density of about 0.2 but with only one species surviving after 20,000 time steps.

The paradox of enrichment appears in simulations with ten algal species under both local and global interactions, and those with 20 and 50 species under local interactions (Fig. 2). Under local interactions, the lowest nutrient level that allows survival of at least one species is *P* > 14.0 (Fig. 3a). Six of the algal species survive at *P* = 14.1 (Fig. 3b). By this, we say that the lowest nutrient level in our simulation is *P* = 14, meaning, anything close to this level is considered low. The highest number of species is ten, occurring at 14.3 nutrient level (Figs. 3a and 3c). After a sudden increase in species diversity from nutrient levels 14 to 14.3, the number decreases immediately from 14.4 and so on, exhibiting the paradox of enrichment (Figs. 2a and 3a). As expected, the equilibrium state is a one-species ecosystem if the nutrient level is 15.5 or higher (Figs. 3a and 3d). Qualitatively the same result is obtained under global interaction (Fig. 2a and 3). The largest number of surviving species is four at *P* = 14.4 (Figs. 4a, 4b and 3c), and it becomes one species when *P* = 14.6 (Fig. 4d), exhibiting a much smaller scale paradox (Fig. 2a).

**Figure 5:**
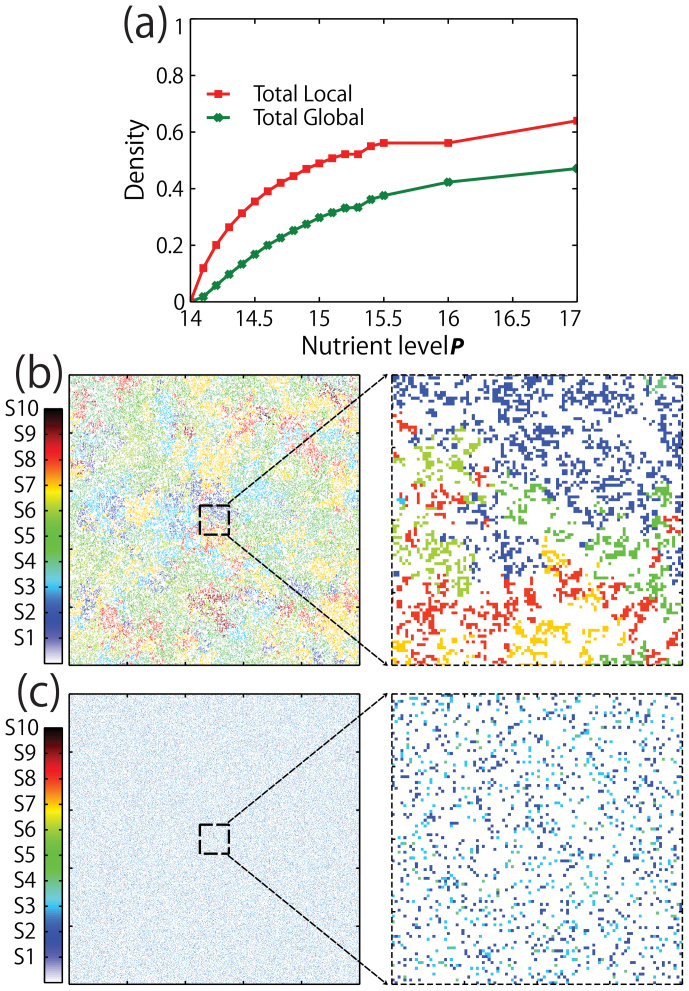
Density profile and snapshots with magnified portions of the ten-species lattice model after 20,000 time steps under local and global interaction. (a) The plot shows the total densities of the ten species under local and global interactions at specific nutrient conditions. The total densities increase along with nutrient concentration. (b) Snapshot of the lattice model at *P* = 14.3 under local interaction exhibits clustering. This is expected since an offspring can only occupy a space that is adjacent to the parent's site. Moreover, the presence of white/empty spaces indicates separation between individuals of the same species resulting to a weak competition. (c) The snapshot of the lattice model at *P* = 14.4 under global interaction also exhibits weak competition because of the empty spaces separating the individuals.

**Figure 6:**
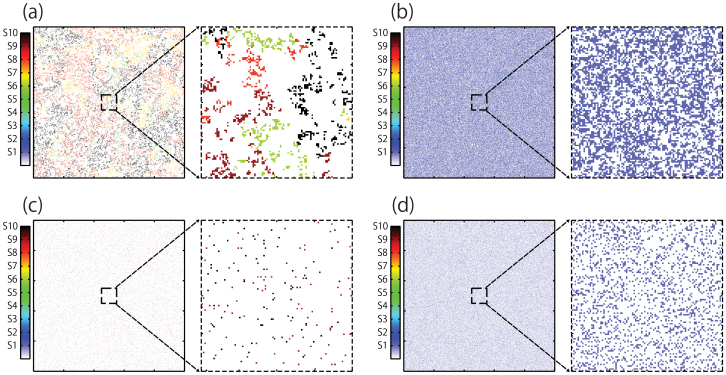
Snapshot of the ten-species lattice model after 20,000 Monte Carlo steps under local and global interaction. Magnified portions of the snapshots are also shown. (a) Snapshot of the lattice model at *P* = 14.1 under local interaction. The system shows clustering of similar species which is expected under local interaction. The wide empty spaces also indicate habitat separation and weak competition. (b) Snapshot of the lattice model at *P* = 15 under local interaction. In this model, two species survived with one of the species having a very low density (Fig. 4d2). (c) Snapshot of the lattice model at *P* = 14.1 under global interaction. The wide white patches also indicate habitat separation and weak competition. (d) Snapshot of the lattice model at *P* = 14.4 under global interaction. After 20,000 Monte Carlo steps, only one species survived.

Under local interactions, interspecific competitions are suppressed by low population density (Fig. 5a) and clumping spatial patterns (Figs. 5b vs. 5c and Fig. 6). The population densities of each species are very small at low nutrient levels where diversity of species is high (Figs. 3b1 and 3c2). In the case of the nutrient level with the highest diversity of species (*P* = 14.3), each steady-state density is less than 0.06 for local interaction (Fig. 3c2 compared with 3d2). Moreover, spatial patterns after simulation exhibit distinctive species clumping (Fig. 5b). Species are clumped and separated by large open/empty areas (white color in Fig. 5b enlarged) so as not to touch each other (Fig. 6a). Thus the negative interactions between species are almost negligible under local interactions. While clumping is solely observed in local interaction, the snapshot under global interaction also shows many empty spaces, indicating the probability of competition is also quite low, since the probability of competition is relatively low (Fig. 5c and Fig. 6c).

Sensitivity analysis was conducted for local interaction to examine if the behavior of the model will change or remain the same by increasing the number of species from ten to twenty and then to fifty. The birth rates were also modified so that the rates are higher even at low nutrient levels. The result of the sensitivity analysis for twenty and fifty species produced similar results (Fig. 2b). Highest species diversity also existed at lower nutrient levels and decreases as nutrient level rises. The fifty-species system reached a maximum of thirty nine species surviving after 20,000 steps at *P* = 14. This peak occurred immediately after the highest nutrient level *P* = 13.9 where none of the species survive. Similarly, the peak of the twenty-species system is at *P* = 14 with all species surviving after the designated time steps. After these peaks, the number of species in both systems decrease drastically as nutrient level rises exhibiting the paradox of enrichment very clearly. However, both systems did not reach a one-species equilibrium after 20,000 time steps (Fig. 2b). The fifty-species system has four species while the twenty-species system has two species after 20,000 time steps at high nutrient levels. Nevertheless, when the final time step is increased to more than 20,000 (40,000 for the twenty-species system and 80,000 for the fifty-species system), competition also leads to one-species survival.

## Discussion

Here we show that the exclusion of all but one species is demonstrated in a simple competition model in a single trophic level. The major contribution here is that we showed simple competition for space in a single trophic level is enough to explain the paradox of enrichment at very high nutrient conditions and coexistence at low nutrient environments without the complexity of the previous models. This is different from the paradox of enrichment via trophic interaction in an important aspect. First, trophic interactions usually involve time delay, because of the predator responses in upper trophic levels^23^. Temporarily, the density of predator species in some trophic levels should increase explosively in the trophic cascades^24^,^25^. Some empirical data on eutrophication seems not agree well with these predictions. We still have to evaluate the trophic interactions to test which hypothesis is valid in a real ecosystem. We thus showed that a very simple competition model for space is enough to exhibit the reduction in species numbers, and more complicated ecosystems (e.g., more than one trophic levels) or extrinsic factors are not necessary to explain the paradox of enrichment. We should have to wait for future empirical studies to test the validity of the proposed mechanisms that eutrophication leads to competitive exclusion via highly increased competition for space.

In our model we assume that the birth rates of phytoplankton species are increasing with the increase in nutrient level. As a consequence, we expect an increase in the possibility of coexistence because of more nutrients for all species^1^. However, we also expect that the increase in nutrients leads to a more competitive ecosystem between species. According to Gause's law of competitive exclusion, two species competing for the same resources cannot coexist if all ecological factors remained constant^20^,^26^,^27^. This proposition is supported by several experimental studies using chemostat^16^,^28^,^29^. Many mathematical studies also demonstrate that the coexistence of many species is extremely unlikely when niches are not separated^7^,^25^,^31^.

Due to these experimental and theoretical results^1^,^7^,^16^,^22^,^26^,^27^,^28^,^29^, many studies proposed to include extrinsic factors such as climate change, immigration, dormancy and spatial heterogeneity of habitats, and chaotic dynamics^30^,^31^,^32^,^33^,^34^,^35^. For example, high disturbance may promote low diversity, since many individual organisms may be located in unsuitable habitats by mixing up the current distribution of phytoplankton^36^,^37^,^38^. Certainly, it is easy to include extrinsic factors in the study of coexistence in ecosystems with two or three species such as the models presented by McCauley and others on plankton diversity^39^. However, considering external factors in ecosystems with more than 10 species is very unlikely due to the extreme sensitivity of tradeoffs between species. Thus, the study of coexistence among large number of species in these systems is inconceivable. Moreover, such external variables tend to be not always relevant to aquatic systems. For instance, the spatial difference in a microscopic habitat is difficult to imagine in aquatic ecosystems, because marine environments are homogenous and the niches of phytoplankton are almost similar. Also, the factor of competition for nutrients in the presence of bacteria is excluded in this study since the existence of bactivores can eliminate the competition^40^.

In our analysis, we consider an ecosystem of several species without external factors, but with vast spatial distributions. A vast space works to avoid competition as niche variation does (Fig. 5b). Moreover, we focus on temporal dynamics of phytoplankton ecosystems in ecological time scales. This is important since the longevity of lakes and ponds, and living organisms, are not infinite but finite^1^,^33^,^41^. Almost all experimental studies are carried out under highly eutrophicated conditions, which is very different from natural poor-nutrient environments. Many cases of red tide and loss of diversity in aquatic ecosystems that appeared recently should be caused by eutrophication due to human activities. This eutrophication or enrichment of aquatic systems would cause intense competition among species, resulting in the loss of species diversity. We suspect that this is the main mechanism of the paradox of enrichment.

The current results are the natural outcomes of species-specific birth rate functions along the nutrient level *P* (Fig. 1). It is natural that the differences in birth rates should be very small under low nutrient levels but become larger when nutrient levels become higher^15^,^16^,^17^,^18^,^19^,^23^. Under low nutrient levels, the difference in photosynthesis among all phytoplankton should be negligible because of physiological limitations. However, under enriched conditions, the growth difference among species should be enlarged. Therefore, one or few species should dominate over all other species. Note that, depending on the limited nutrition, the dominant species may be different^19^,^23^. However, the results are highly robust, because high nutrient condition does not only increase birth rates but also magnifies the variability of birth rates between species (Fig. 1). Most importantly, high nutrient conditions lead to severe competition for space (lattice sites), resulting in the extinction of all but one dominant species (Figs. 3d1 and 4d1).

In our study, the dynamics of the population in terms of local and global interactions are different. However, simulation showed that both systems under local and global interactions produced similar results. At high nutrient levels, a superior species emerges at the expense of the others (Figs. 3d and 4d). However, high diversity can be achieved at low nutrient conditions. Also, at low nutrients, species tend to stay at low but almost similar densities without any species standing out among the others (Figs. 3c and 4c). The temporal population dynamics at low nutrient levels becomes extremely slow because of low growth rates. Hence, in ecological times scale, the coexistence of species is achieved when nutrient availability is scarce, even though the mathematical expectation in the infinite time horizon is the exclusion of all but one superior species.

The transition from coexistence to a one-species-dominated phase or vice versa has been observed in many lattice Lotka-Volterra-like models^10^,^11^,^25^,^42^,^43^,^44^,^45^,^46^,^47^. However, these studies considered different complicated dynamics such as predation and diffusion which is not considered in the current lattice competition model (multiple contact process)^48^,^49^. We also consider the metastable (quasi-stationary) states in the ecological time scale^1^,^25^,^33^,^47^,^48^,^49^, unlike the equilibrium states in the infinite future considered in most of these complex models^cf. ref. 47^. Because the mean decay time of metastable states typically grows exponentially with the lattice size^48^,^49^, the current results should be realistic for the prediction of lake and pond ecosystems.

Rosenzweig^1^,^50^ had shown that eutrophication increase the stability of ecosystems resulting in the paradox of enrichment via trophic interactions. Here we demonstrate that the paradox of enrichment can also be induced via competitive interactions among species in a single trophic level. From natural observations^7^,^8^, the paradox of enrichment in phytoplankton in lakes and ponds fits more to that of a single trophic level, since no distinctive change in trophic interactions is observed, but instead we find an increase in a single phytoplankton species. In contrast, the paradox of enrichment via trophic interaction may be dominated in animal and insect communities in which predation and/or parasitism is a major factor of population fluctuations. The instability induced by enrichment in predator-prey or host parasite systems may be important in many terrestrial ecosystems^1^,^38^. In contrast, in aquatic ecosystems, enrichment induces competition for space among phytoplankton species^18^,^31^.

Finally, we should note that our model shows a mechanism of competition avoidance. It is well-known that individual species easily become extinct in a finite lattice^25^. However, our results show that at low nutrient levels, extinction seems to be delayed significantly, due to extremely low or lack of competition. Under local interactions, the clumping of species in the snapshot pattern at low nutrients indicates much lower level of competition between species, but higher competition between individuals of the same species (Fig. 5b). This is because that an offspring (new cell) occupies a site next to the parent under local interactions. This results to the segregation of habitats where species can coexist without actual competition. This implies that the low mobility of individual phytoplankton is a key factor to promote coexistence in low nutrient conditions. Mobility of phytoplankton in freshwater ecosystems such as lakes and ponds is limited because of the viscosity of water and because water movement is only affected by wind strength and size of the body of water^51^. In reality, the mobility of phytoplankton in lakes and ponds should be something in between local and global interactions. Note that the results of global interactions are almost the same with local interactions', only with lesser degrees^52^,^53^,^54^,^55^. Thus we conclude that the paradox of enrichment of phytoplankton in lakes and ponds are the result of increased competition between species due to the enhancement of plankton growth by increased nutrients.

## Methods

### Lattice model

We consider a competitive system of ten phytoplankton algal species (i = 1,2,…,*N* = 10) and apply a two-dimensional lattice (1000 × 1000), since phytoplankton species scatter near the water surface and compete for sunlight. Each lattice patch is either occupied by *i* species (*X_i_*) or empty (*O*) (one individual per patch). Overall dynamics are as follows: 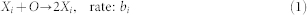

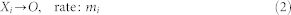
where *X_i_* is an individual of species *i* of *N* species. The parameters *b_i_* and *m_i_*, denote the birth and death rates, respectively. The birth rate *b_i_* of species *i* is computed using the following logistic equation: 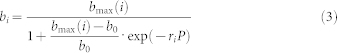
with parameters computed as follows 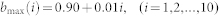

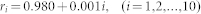

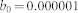
where *b*_max_(*i*) is the maximum birth rate of the *i*th species when the nutrient level *P* goes to infinity. The simulation is carried out according to the neighborhood process where local interaction occurs between the location of an individual species and the four adjacent lattice sites (right, left, up and down)^10^,^14^,^32^.

Equation 3 is imitating Monod or Monod-like equations for higher nutrient levels (*P* ≥ 14), where growth rates becomes positive. Because birth rates cannot be negative when nutrient level is too low, birth rate function should be a sigmoid curve starting near the origin (0, 0). In the current equation, the plot starts at (0, *b*_0_), where *b*_0_ = 0.000001 (Fig. 1a). In our analyses, we only look at the dynamics at *P* = 14 or larger, so that at least one species survive at the lowest nutrient level.

The birth rates of ten species are set as follows.

1. At high *P*, the birth rates are largely different.
2. At low *P* with positive density, the values of bi are very close.
3. Species with high birth rate at high *P* has low birth rate at low *P* (tradeoff of birth rate along nutrient level *P*).

These conditions represent the features of birth rate in phytoplankton^15^,^19^,^23^,^30^,^56^,^57^.

The mortality rate is kept constant at *m_i_* = 0.3 and 0.5 for local and global interactions, respectively. We use a higher mortality rate for the global interaction since it is more efficient in terms of reproduction than the local interaction^10^,^12^,^13^,^33^. This difference in efficiency is shown in the study by Miyazaki et al.^33^ using comparative parameter conditions where similar birth and mortality rate were used in the simulation under local and global interaction. They showed that the extinction threshold for positive density is lower under global interaction, given similar mortality rates. To balance this difference in efficiency and to close to gap between global and local interactions, we set a higher mortality rate for the global interaction (please refer to Figure 1b). Here the extinction thresholds for positive density are set approximately equal for both interactions. We believe that a similar threshold for positive density at lower nutrient levels for both interactions will make a better comparison of the diversity at specific nutrient levels. The extinction thresholds are *b* = 0.53 and *b* = 0.54 for local with *m_i_* = 0.3 and global with *m_i_* = 0.5, respectively. We should also note that growth rate is the key factor in these lattice models, but not individual birth rates and mortality rates. For example, if we set both mortality rates equal, the realized growth rate of the local interaction become much smaller than that of the global interaction.

For global interactions, two lattice sites are chosen in random from the whole lattice. In this case, the population dynamics of the system is defined by the mean-field theory. Let *x_i_* be the overall density of species *i*. Since the probability of finding the individual *X_i_* becomes equal to the overall density of *X_i_*, we the following dynamics 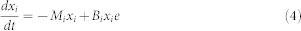
where *i* = 1,2,…,*N* and *e* is the density of empty site (*O*). Note that 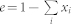.

The first and second terms on the right site of Eq. 4 denote death and birth processes, respectively. For instance, suppose we consider the cases of *N* = 1 and *N* = 2. For a single species system, *N* = 1, Eq. 3 becomes the logistic equation defined by: 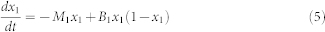
The non-zero steady-state density for this equation is given by 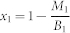.

On the other hand, in the two-species system, *N* = 2, Eq. 3 can be written as 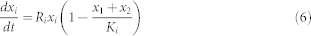
where the parameters *R_i_* and *K_i_* are defined as follows: 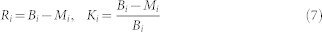
Equation 6 is known as the Lotka-Volterra competition model whose results are well documented. Lastly, stationary states are classified into four classes, depending on the values of the parameters. Namely, given species *X* and *Y*, (1) both *X* and *Y* coexist, (2) only *X* persists, (3) only *Y* survives, and (4) both species become extinct. The condition for the coexistence of this system is defined by: 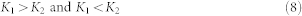
The above two relations are not satisfied simultaneously. Hence, at least 1 species becomes extinct. In general, in the case of *N* > 2, we can show that the coexistence of at least 2 species is impossible^10^.

### Simulation procedure

The simulation procedures of local interaction for each nutrient level *P* are as follows:

1. Phytoplankton cells are distributed randomly over some of the square-lattice points in such a way that each point is occupied by only one individual of a certain species, if the point is occupied.
2. The reaction process is performed in the following manner.
  - To perform the single body reaction (2), choose one square-lattice point randomly. If the point is occupied by species *i*, then change it to *O* with probability *m_i_*. No change otherwise.
  - Next, perform the two-body reaction (1) by selecting one point randomly and specify one adjacent point. Here, the adjacent site is set as the Neumann neighbors (up, down, left or right). If the selected pair are *X_i_* and *O*, respectively, then the latter point will become *X_i_* with probability *b_i_*. Otherwise, the points remain unchanged. Here, we utilize periodic boundary conditions.
3. Repeat step 2 *L* × *L* times, where *L* × *L* is the total number of the square-lattice sites. Here we set *L* = 1000. This step is called a Monte Carlo step.
4. Repeat step 3 for a specific length (20,000 Monte Carlo steps).

This simulation procedure is repeated for each nutrient level *P* with increments of 0.1.

Furthermore, the simulation process for global interactions is almost identical except for the two-body reaction. In step 2, letter b), we select two lattice points in random and independent of their location.

## Author Contributions

J.Y., J.M.T., T.T. and K.T. conceived the study. J.M.T. and K.T. built the program and J.M.T. run the simulations with the help of H.I., S.M., and T.U. S.K., T.T., M.P.N., B.E.C. and Y.S. provided the information about eutrophication in aquatic systems. J.M.T. and J.Y. wrote the manuscript, and all authors contributed to finalizing the manuscript.
